# Epitaxial growth of highly-crystalline spinel ferrite thin films on perovskite substrates for all-oxide devices

**DOI:** 10.1038/srep10363

**Published:** 2015-06-01

**Authors:** Jarrett A. Moyer, Ran Gao, Peter Schiffer, Lane W. Martin

**Affiliations:** 1 Department of Physics, University of Illinois at Urbana-Champaign, Urbana, IL 61801; 2 Materials Research Laboratory, University of Illinois at Urbana-Champaign, Urbana, IL 61801; 3 Department of Materials Science and Engineering, University of Illinois at Urbana-Champaign, Urbana, IL 61801; 4 Department of Materials Science and Engineering, University of California, Berkeley, Berkeley, CA 94720; 5 Materials Science Division, Lawrence Berkeley National Laboratory, Berkeley, CA 94720.

## Abstract

The potential growth modes for epitaxial growth of Fe_3_O_4_ on SrTiO_3_ (001) are investigated through control of the energetics of the pulsed-laser deposition growth process (via substrate temperature and laser fluence). We find that Fe_3_O_4_ grows epitaxially in three distinct growth modes: 2D-like, island, and 3D-to-2D, the last of which is characterized by films that begin growth in an island growth mode before progressing to a 2D growth mode. Films grown in the 2D-like and 3D-to-2D growth modes are atomically flat and partially strained, while films grown in the island growth mode are terminated in islands and fully relaxed. We find that the optimal structural, transport, and magnetic properties are obtained for films grown on the 2D-like/3D-to-2D growth regime boundary. The viability for including such thin films in perovskite-based all-oxide devices is demonstrated by growing a Fe_3_O_4_/La_0.7_Sr_0.3_MnO_3_ spin valve epitaxially on SrTiO_3_.

Continued advances in the growth and fabrication of complex-oxide thin films have led to the creation of an ever-increasing number of functional devices. All-oxide devices, such as the strain-driven magnetoelectrics (*i*.*e*., BaTiO_3_-CoFe_2_O_4_ and BiFeO_3_-CoFe_2_O_4_ composites and La_0.67_Sr_0.33_MnO_3_-BaTiO_3_ heterostructures)^1^,^2^,^3^, charge-driven magnetoelectrics (*i*.*e*., La_0.8_Sr_0.2_MnO_3_-PbZr_0.2_Ti_0.8_O_3_ and La_0.67_Sr_0.33_MnO_3_-BaTiO_3_ heterostructures)^4^,^5^,^6^, La_0.67_Sr_0.33_MnO_3_-based magnetic tunnel junctions^7^,^8^,^9^, and CoFe_2_O_4_-, NiFe_2_O_4_-, MnFe_2_O_4_-based spin filters^10^,^11^,^12^,^13^, show much promise for technological advancements. Key to many such devices has been the development of heteroepitaxial growth of different oxide materials on top of one another^14^,^15^. Despite a diverse range of complex oxide materials, however, the majority of work in this area has focused on perovskite-based materials. This is due to the multitude of properties exhibited by this class of materials, as well as the ease of growth of multi-layer systems that results from the continuous chemical structure and relatively narrow range of lattice parameters presented by these materials^16^,^17^. Strikingly, researchers have largely neglected a huge range of functional complex oxides with other crystal structures – some possessing more desirable properties and better performance than their perovskite counterparts. For instance, one drawback to the perovskites is that many of the novel properties that make them attractive to researchers are only accessible below room temperature; this is nicely exemplified by ferromagnetism, where very few perovskite systems exhibit ferromagnetism above room temperature (*e*.*g*., *T*_c_ ~ 370 K for La_0.7_Sr_0.3_MnO_3_)^18^. In order for the oxide electronics field to mature to the point where all-oxide devices become viable alternatives to current electronic devices, more research needs to be focused on room temperature devices, which will necessarily require materials with other crystal structures.

The spinel ferrites comprise an important class of candidate materials that could serve as the magnetically-active layer in room temperature, all-oxide devices. These materials, with composition *TM*Fe_2_O_4_ where *TM* is a transition-metal cation, are ferrimagnets that can retain their magnetism to above 800 K (*e.g.*, *T*_c_ = 858 K for Fe_3_O_4_)^19^. They crystallize in the spinel crystal structure, where 2/3 of the cations are octahedrally coordinated and 1/3 of the cations are tetrahedrally coordinated; the octahedral and tetrahedral cations are antiferromagnetically aligned, which gives rise to ferrimagnetism. In addition to remaining magnetic to well above room temperature, the resistivity of the spinel ferrites can be tuned by over four orders of magnitude through cation substitution without greatly affecting *T*_c_^20^,^21^,^22^. The parent compound of the spinel ferrites, Fe_3_O_4_, has been widely studied in the bulk due to its so-called Verwey transition, which is a structural and charge and orbital ordering phase transition at ~120 K that results in a two orders of magnitude increase in the resistivity^23^,^24^,^25^,^26^,^27^. In thin films, the Verwey transition decreases in temperature and broadens as the film thickness is decreased, which has been hypothesized to be due to both epitaxial strain effects and the increased density of structural domains due to anti-phase boundaries^28^,^29^. These boundaries are known to form in spinels when they are grown on substrates that have lattice constants that are roughly half of the spinel crystal structure, such as the perovskites or MgO, resulting in structural domains that are out-of-phase with each other. Thus, the structural quality of thin-film Fe_3_O_4_ can be correlated to the sharpness of the Verwey transition and the temperature at which it occurs.

Ultimately, the desired functionality of an all-oxide device would likely require that one can incorporate high-temperature magnetic spinel ferrites with perovskites that display a range of functionalities, *e*.*g*., dielectric, piezoelectric, ferroelectric, metallic, magnetic, etc. Such combinations of spinel ferrites and perovskites have already proven to be important in the case of strain-driven magnetoelectric devices, where they are grown either within a piezoelectric matrix or epitaxially on top of a piezoelectric substrate^1^,^2^,^30^,^31^. In both of these cases, good epitaxial growth is not critical as the functionality of the spinel ferrite is derived from the bulk-like magnetostriction applied through the piezoelectric material. For some other applications (*e.g.*, field effect devices), it would be necessary to grow high-quality, thin epitaxial layers of the spinel ferrites on top of perovskites. Much work has already been completed attempting to grow spinel ferrites on perovskite substrates; such growth is not easy, however, because of a large lattice mismatch between the spinels and perovskites, on the order of −7%, and because of the tendency for the spinels to grow in an island growth mode on top of perovskites^32^,^33^. For Fe_3_O_4_ thin films (<100 nm), this results in films that have degraded physical properties (*e*.*g*., decreased magnetic moment) as compared to films grown on MgO, which due to a small lattice mismatch of ~0.3% with the spinel ferrites, allows for coherently strained growth of high-quality thin films^20^,^28^,^34^,^35^,^36^,^37^.

In this work, we explore how control of the energetics of the growth process can affect the growth mode and physical properties of spinel ferrite thin films grown on perovskite substrates and films. Specifically, we examine the prototypical spinel ferrite, Fe_3_O_4_, and the perovskites SrTiO_3_ and La_0.7_Sr_0.3_MnO_3_. By monitoring the growth *in situ* with reflection high-energy electron diffraction (RHEED), we find that Fe_3_O_4_ can grow epitaxially on perovskites in one of three distinct growth modes that we label “2D-like,” “3D-to-2D,” and “island.” Films grown in the 2D-like and 3D-to-2D growth modes tend to be under partial in-plane compressive strain and have atomically smooth surfaces, while films grown in the island growth mode are nearly fully relaxed and terminated by islands. By growing films with multiple combinations of laser fluence and substrate temperature, we are able to map out a phase diagram of the different growth modes. As the energetics of the growth process are increased, the growth mode changes from 2D-like to 3D-to-2D to island. Transport measurements reveal that films grown in a 3D-to-2D growth mode have the lowest resistivities, while magnetometry measurements demonstrate that the films grown in this growth mode possess the sharpest Verwey transition, sharpest magnetization reversal, and largest magnetic moment. Using the information gained from the thin-film growths, a spin valve of La_0.7_Sr_0.3_MnO_3_/Fe_3_O_4_ was created that displayed distinct features when the La_0.7_Sr_0.3_MnO_3_ and Fe_3_O_4_ layers switched from anti-parallel to parallel spin alignments, demonstrating the suitability of these films for device applications.

## Results and Discussion

### Structural characterization

**Figure 1:**
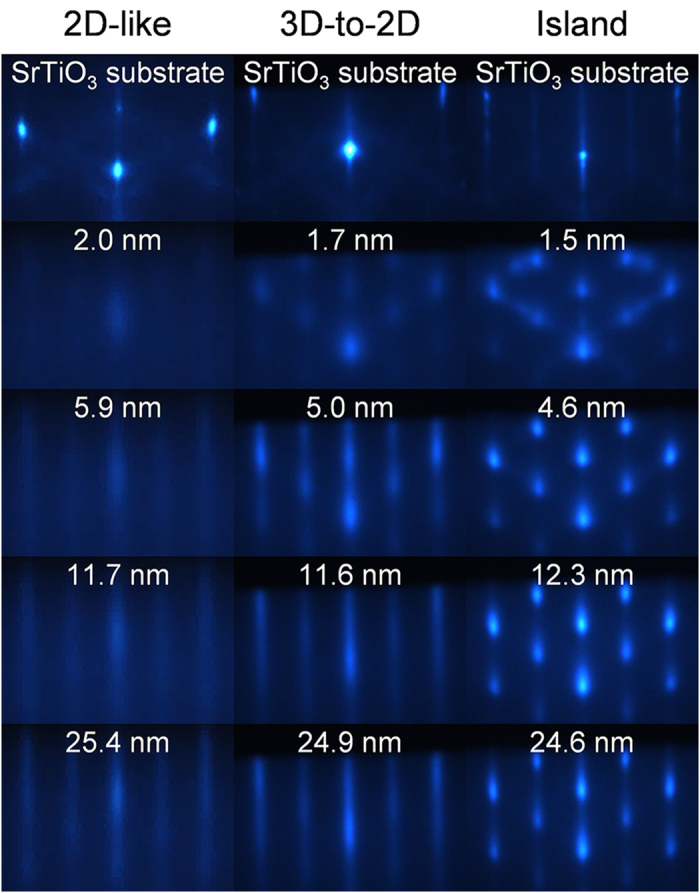
RHEED patterns taken along the [110] crystal axis during growth for Fe_3_O_4_ films grown in the 2D-like (2.3 J/cm^2^, 400 °C), 3D-to-2D (1.5 J/cm^2^, 500 °C), and island (1.5 J/cm^2^, 600 °C) growth modes on SrTiO_3_ (001) substrates. The thickness of the film for each RHEED pattern is noted.

The effects of the energetics of the growth process were monitored *in situ* with RHEED, which revealed that Fe_3_O_4_ can grow epitaxially on SrTiO_3_ in one of three different growth modes identified above [Fig. 1 and Table 1]. For all three growth modes, we observe a change in the location of the RHEED streaks within the first 1 nm of growth, signifying a relaxation of the strain due to the formation of defects at the substrate-film interface. 2D-like growth is characterized by diffuse RHEED streaks at the beginning of the growth (<5 nm film thickness) that continually become sharper throughout the duration of the growth, whereas island growth is characterized by RHEED patterns that are spotted throughout the entirety of growth. The third growth mode, which we are calling 3D-to-2D, is not one of the canonical growth modes, but has been observed previously in GaN/Al_2_O_3_ (0001)^38^, SrRuO_3_/SrTiO_3_ (111)^39^, and Fe_3_O_4_/BaTiO_3_ (001)^40^. These films begin growing in an island growth mode as evidenced through spotted RHEED patterns (<10 nm film thickness), but, after a critical thickness, the islands merge together, and the films continue to grow in a 2D growth mode as evidenced by streaky RHEED patterns. We define the critical thickness as the thickness at which the intensity oscillation in a line scan along the specular diffraction streak vanishes, signifying that the islands have coalesced and that the film surface is smooth.

The variations in the RHEED patterns between films grown in the different growth modes demonstrate the differences, not only in the morphology, but also in the nature and the density of defects that form to relieve the strain. The initial diffuse RHEED patterns for the films grown in the 2D-like growth mode signify that these films have a greater density of dislocations forming to relieve the strain compared to the other two growth modes. This is due to the fact that the 3D-to-2D and island growth modes also relieve the strain though island formation, which is common for many spinel/perovskite couples. The initial growths of these modes are similar to each other, signifying that the number and density of defects within them are similar to each other.

**Figure 2:**
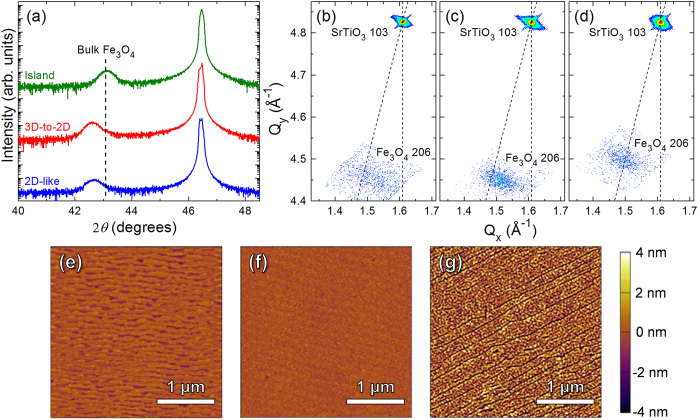
(**a**) 2*θ-ω* scans of the Fe_3_O_4_ 004-diffraction condition for Fe_3_O_4_ films grown in the 2D-like (2.3 J/cm^2^, 400 °C), 3D-to-2D (1.5 J/cm^2^, 500 °C), and island (1.5 J/cm^2^, 600 °C) growth modes. Corresponding reciprocal space maps about the Fe_3_O_4_ 206-diffraction condition for Fe_3_O_4_ films grown in the (**b**) 2D-like, (**c**) 3D-to-2D, and (**d**) island growth modes and AFM images for Fe_3_O_4_ films grown in the (**e**) 2D-like, (**f**) 3D-to-2D, and (**g**) island growth modes.

We further analyzed the structure of the films with X-ray diffraction measurements. Wide angle scans reveal single-phase films that are, with some exceptions noted below, 00*l*-oriented. Additional close inspection about the Fe_3_O_4_ 004-diffraction condition [Fig. 2(a)] suggests that films grown in the island growth mode have fully relaxed structures whereas those grown in the 3D-to-2D and 2D-like growth modes exhibit out-of-plane lattice parameters consistent with the presence of residual in-plane compressive strain. Reciprocal space maps about the SrTiO_3_ 103- and Fe_3_O_4_ 206-diffraction conditions were used to further investigate the in-plane strain [Figs. 2(b)-(d)]. These mappings confirm that the films grown in the 2D-like and 3D-to-2D growth modes have residual in-plane strains as high as -0.6% (this is much smaller than the −7% strain that would occur for a coherently strained film), while the films grown in an island growth mode are essentially relaxed [Table 1]. Additionally the 206-diffraction condition for the films grown in a 2D-like growth mode is more diffuse than the films grown in the 3D-to-2D and island growth modes, which is consistent with the diffuse RHEED patterns seen in the initial stages of film growth. Characterization of the surface morphology of the films grown in the different growth modes reveals films grown in the 2D and 3D-to-2D growth modes possess atomically smooth surfaces with atomic steps, while films grown in the island growth mode show markedly rougher surfaces consistent with island formation [Fig. 2(e)-(g), Table 1].

**Figure 3:**
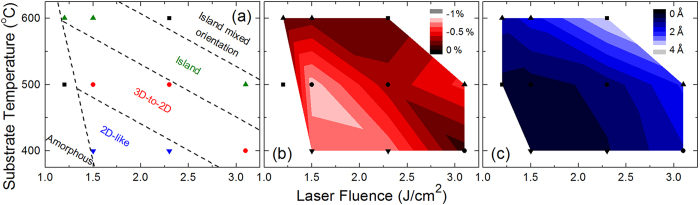
The effects of the energetics of the growth process. These plots demonstrate how substrate temperature and laser fluence affect (**a**) growth mode, (**b**) in-plane epitaxial strain, and (**c**) surface roughness as a function of substrate temperature and laser fluence. The dotted lines are provided as approximate growth mode regime boundaries as a guide to the eye.

In pulsed-laser deposition thin-film growth, the energetics of the growth process can be controlled by adjusting the laser fluence and/or the substrate temperature. By growing numerous films with different laser fluences and substrate temperatures, we were able to map how the energetics of the growth process affect the growth mode, in-plane strain, and surface roughness [Fig. 3(a)–(c), respectively]. While the growth mode can be changed through both substrate temperature and laser fluence, it is easier to switch between growth modes through changes in substrate temperature. As the energetics of the growth process are made either very large or small, Fe_3_O_4_ no longer grows fully epitaxial; large energies result in a mixed (001)/(111)-orientation and small energies result in amorphous films. Additionally, the critical thickness (*i.e*., when the film switches over from island to 2D growth) for the 3D-to-2D growth mode can be decreased (increased) by decreasing (increasing) the energy during growth. In other words, by moving closer to the 2D-like (island) growth mode regime the critical thickness is decreased (increased). The lowest critical thickness we observed in the 3D-to-2D growth regime where the sample became atomically flat was just 10 nm for a film grown at 1.5 J/cm^2^ and 400 °C. As noted above, such a 3D-to-2D growth mode has been observed previously for Fe_3_O_4_, but a thickness of ~50 nm was required for the islands to fully converge^40^. By precisely controlling the energetics of the growth process, we have shown that one can achieve atomically flat films as thin as 10 nm for films grown in a 3D-to-2D growth, which is near the thickness required for incorporating a thin film in a field-effect device.

Comparing the in-plane strain diagram [Fig. 3(b)] with the growth mode diagram [Fig. 3(a)] indicates that the strain does not change in a linear manner with growth energy. The largest in-plane strains occur around the 2D-like/3D-to-2D growth regime boundary. Recall that in this system, the strain can be relieved through island formation. In the 3D-to-2D growth regime, this is initially how the strain is relieved. But as the film thickness increases, the surface energy starts to dominate over the interfacial energy driving the film towards 2D growth and the coalescence of the islands, which quenches the mechanism for strain relaxation. Hence, the thinner the film growth required for the transition from island growth to 2D growth, the larger the residual in-plane strain, which should occur at the 2D-like/3D-to-2D growth regime boundary. This is exactly what we observe for films grown in the 3D-to-2D growth mode: films in which the islands coalesced at 10, 18, and 22 nm had residual in-plane strains of −0.6%, −0.2% and −0.01%, respectively. As the energetics of the growth process decrease from this boundary (*i.e.*, transitioning into the 2D-like growth mode regime), the strain decreases slightly. This is possibly due to a decrease in crystallinity (increase in defect density) as the films approach the amorphous film growth regime; this is supported by the diffuse RHEED patterns during the initial stage of 2D-like growth. Conversely, as the energy increases from the 2D-like/3D-to-2D growth regime boundary (*i.e.*, transitioning through the 3D-to-2D growth mode regime towards the island growth mode regime), the strain also decreases, and eventually the films become fully relaxed in the island growth mode regime. The diagram of the surface roughness is remarkably similar to that of the in-plane strain, with the films grown in the 2D-like and 3D-to-2D growth regimes behaving similarly with atomically smooth surfaces, while the films grown in the 3D growth regime are terminated in islands and exhibit increased roughness.

From the collective RHEED, X-ray diffraction, and AFM measurements a complete picture emerges on how the structure and growth mode of Fe_3_O_4_ thin films grown on SrTiO_3_ evolve as the energetics of the growth process are varied. High-energy growth conditions (*i.e.*, high temperature, high fluence) result in spinel ferrite films that are island terminated, which is well-known and problematic for including these materials in oxide heterostructures and devices. Previous studies have shown that lowering the growth energy can result in smoother surfaces for the spinel ferrites^32^,^33^, but they provided no indication of the two growth regimes that can produce atomically flat films. In addition, we have shown that films grown at the 2D-like/3D-to-2D growth regime boundary do not fully relax, but retain a considerable amount of in-plane compressive strain.

### Transport and magnetic properties

While being able to grow atomically-smooth, epitaxial films of the spinel ferrites on perovskites is important for incorporating the spinels into devices, they must also retain the desired bulk-like physical properties to be useful in such devices. In our work we found that films in the same growth mode regime display many of the same electrical transport and magnetic properties. For brevity, we therefore will focus on the transport and magnetic properties of the best films from each growth mode regime (2D: 2.3 J/cm^2^, 400 °C; 3D-to-2D: 1.5 J/cm^2^, 500 °C; island: 1.5 J/cm^2^, 600 °C) to illustrate the differences between the growth mode regimes.

**Figure 4:**
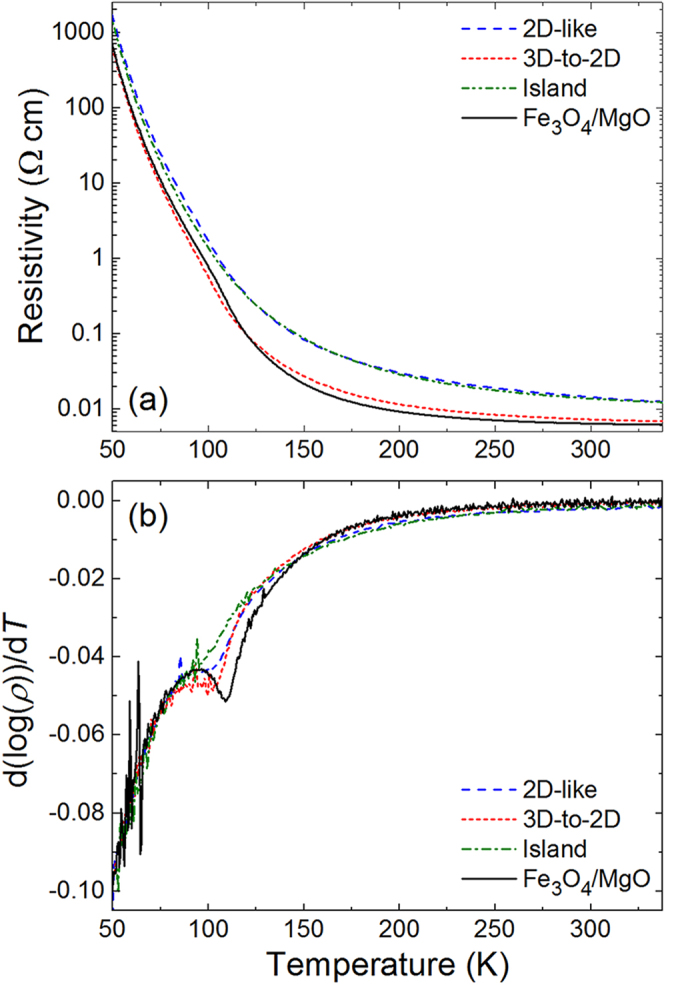
(**a**) Resistivity vs. temperature data for Fe_3_O_4_ films that are grown in 2D-like (2.3 J/cm^2^, 400 °C), 3D-to-2D (1.5 J/cm^2^, 500 °C), and island (1.5 J/cm^2^, 600 °C) growth modes. Data from a Fe_3_O_4_ film grown on MgO is shown for comparison. (**b**) First derivatives of log(*ρ*) for the same samples as shown in (**a**).

The transport properties of these films are examined by comparing their resistivity as a function of temperature with a coherently strained Fe_3_O_4_ film grown on MgO (001) [Fig. 4(a)]. Recall that the lattice mismatch between the Fe_3_O_4_ and the SrTiO_3_ (001) substrate is –7% while that between Fe_3_O_4_ and MgO (001) substrate is only 0.3%. The films grown in the 2D-like and island growth modes have almost identical resistivities for the entire temperature range, with there being a slight increase in the resistivity of the film grown in the 2D-like growth mode compared to the film grown in the island growth mode below the Verwey transition (~120 K). Both of these films, however, have resistivities that are higher than that of the film grown in a 3D-to-2D growth mode; these larger resistivities likely originate from the islands in the film grown in the island growth mode and the reduced crystallinity for the film grown in the 2D-like growth mode. It is interesting to note that the film grown in the 3D-to-2D growth mode has a resistivity close to that of the Fe_3_O_4_ film grown on MgO. The film grown on MgO has a much sharper Verwey transition than the films grown on SrTiO_3_, which also all show a slight decrease in the Verwey transition temperature. The sharpness and temperature of the transition can be seen more readily by plotting the derivative of the log of the resistivity [Fig. 4(b)]. This resistivity data signifies that all three of these films have a higher density of structural defects than the film grown on MgO and possibly a decrease in domain size, with the Fe_3_O_4_ film grown in the 3D-to-2D growth mode being nearly the same (at least in its transport properties) as the film grown on MgO. This is rather surprising considering the large difference in chemical structure and lattice parameter between the spinel ferrite and perovskite materials.

**Figure 5:**
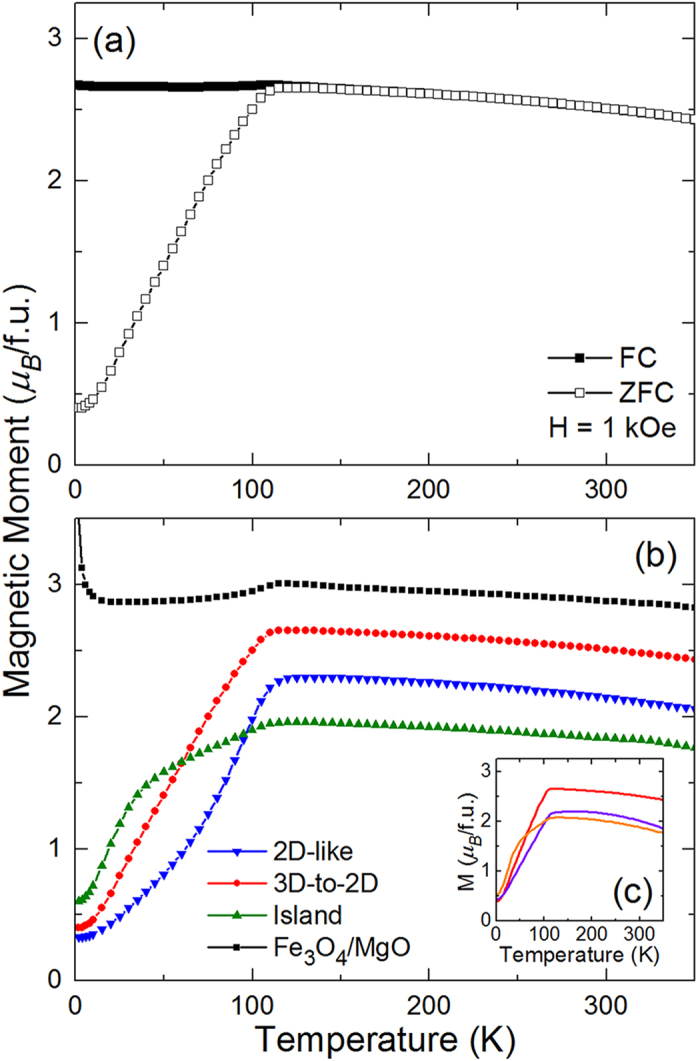
(**a**) FC and ZFC *M*-*T* curves for a Fe_3_O_4_ film grown in a 3D-to-2D growth mode (1.5 J/cm^2^, 500 °C). (**b**) ZFC *M*-*T* curves for Fe_3_O_4_ films grown in the 2D-like (2.3 J/cm^2^, 400 °C), 3D-to-2D (1.5 J/cm^2^, 500 °C), and island (1.5 J/cm^2^, 600 °C) growth modes. Data from a Fe_3_O_4_ film grown on MgO is shown for comparison. (**c**) ZFC *M*-*T* curves for three different Fe_3_O_4_ films grown in a 3D-to-2D growth mode (red: 1.5 J/cm^2^, 500 °C, purple: 2.3 J/cm^2^, 500 °C, orange: 3.1 J/cm^2^, 400 °C).

In addition to being observed in the transport measurements, the Verwey transition can also be observed as a sharp change in the magnetization as a function of increasing temperature. This is demonstrated for the film grown in a 3D-to-2D growth mode with measurements being made in a 1 kOe field after both field cooling (FC) in 1 kOe and zero field cooling (ZFC) [Fig. 5(a)]. While there is only a slight cusp in the FC curve at 120 K, there is a long, steady increase in the magnetic moment of the ZFC curve up to ~120 K, where it flattens out and decreases slowly with further increasing temperature. It is clear from these measurements that the films have a spontaneous magnetization at temperatures well above room temperature, since the magnetization only decreases slightly from its value at the Verwey transition at the temperature limit of the measurement (350 K). The loss of a distinct Verwey transition in the film grown in an island growth mode is clearly seen when the ZFC curves of films from the three growth modes are compared [Fig. 5(b)]. The films grown in 3D-to-2D and 2D-like growth modes both have clear Verwey transition in their ZFC curves, while the film grown in the island growth mode has a ZFC curve that has an initial quick rise up to ~35 K that gradually increases up to 120 K. This is in agreement with the transport measurements that showed a weaker Verwey transition for the film grown in the island growth mode compared to the films grown in the other growth modes.

**Figure 6:**
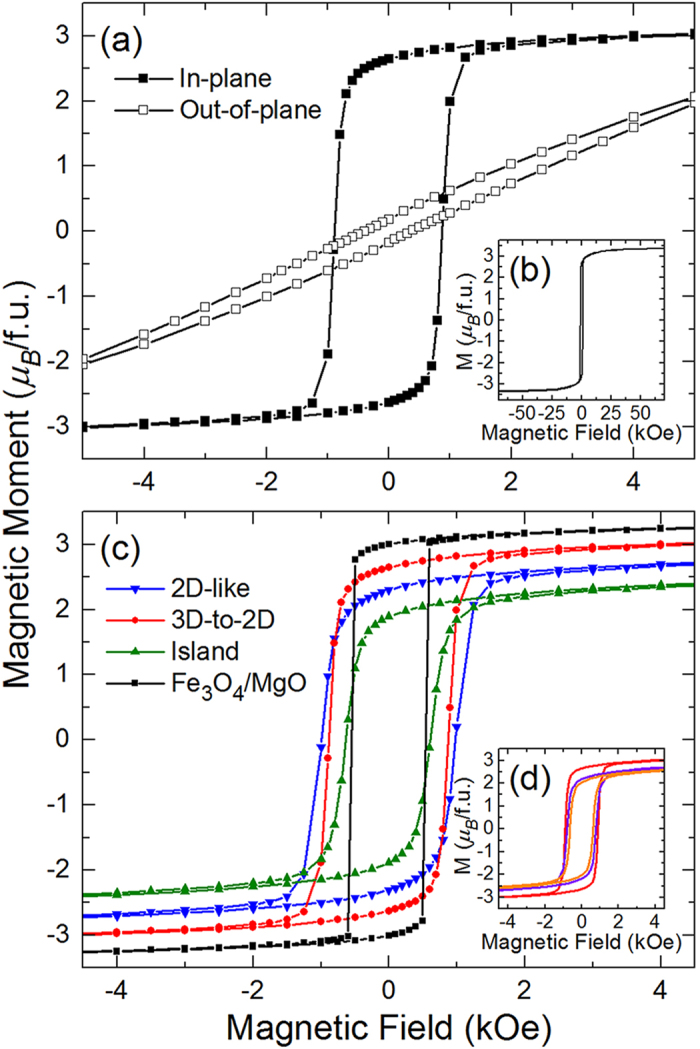
(**a**) In-plane and out-of-plane *M*-*H* loops for a Fe_3_O_4_ film grown in a 3D-to-2D growth mode (1.5 J/cm^2^, 500 °C). (**b**) In-plane *M*-*H* loop for same film (**a**) measured up to 7T. (**c**) In-plane *M*-*H* loops for Fe_3_O_4_ films grown in the 2D-like (2.3 J/cm^2^, 400 °C), 3D-to-2D (1.5 J/cm^2^, 500 °C), and island (1.5 J/cm^2^, 600 °C) growth modes. Data from a Fe_3_O_4_ film grown on MgO is shown for comparison. (**d**) In-plane *M*-*H* loops for three different Fe_3_O_4_ films grown in a 3D-to-2D growth mode (red: 1.5 J/cm^2^, 500 °C, purple: 2.3 J/cm^2^, 500 °C, orange: 3.1 J/cm^2^, 400 °C).

The magnetic properties of the films grown with each growth mode were further analyzed by measuring the magnetic moment as a function of magnetic field (*M*-*H* loop). Note that the diamagnetic response of the substrate, significant for higher fields, was subtracted for all data shown. Films grown with each growth mode all had an in-plane easy axis, as determined from measuring the in-plane and out-of-plane *M*-*H* loops at 50 K [characteristic data for a film grown in the 3D-to-2D growth mode is provided, Fig. 6(a)]. As is common in the spinel ferrites, the magnetization did not saturate for any of the films and is reduced from the bulk value of 4.1 μ_B_/f.u. This reduced moment has previously been attributed to formation of anti-phase boundaries within the Fe_3_O_4_ film [Fig. 6(b)]^28^,^35^. These boundaries result in the formation of antiferromagnetic superexchange interactions that are not present in the spinel crystal structure. Such interactions are stronger than the magnetic interactions that are native to the spinel ferrites, leading to magnetic moments that require very large fields to saturate (>7 T)^35^. As the film thickness decreases, the domain size decreases^29^, which leads to a larger density of anti-phase boundaries and large reductions in the magnetic moment for thin films – consistent with our data^28^,^41^.

Further differences in the magnetic properties between the films grown with different growth modes can be observed when comparing the 50 K in-plane *M*-*H* loops for each sample [Fig. 6(c)]. The films grown in the 3D-to-2D growth mode have the largest magnetic moment, followed by those grown in the 2D-like and island growth modes, respectively. The films grown in the 3D-to-2D growth mode also have the sharpest magnetization reversal in their in-plane *M*-*H* loop, indicating an enhanced long-range order compared to the films grown in the 2D-like and island growth modes. The coercive fields for the films grown in the 3D-to-2D and 2D-like growth mode are roughly the same and are both larger than that of the film grown in the island growth mode. While the magnetometry measurements support the conclusions of the transport measurements that films grown in the 3D-to-2D growth mode have superior physical properties compared to films grown in the 2D-like and island growth modes, the magnetometry measurements make a finer distinction amongst the other films in that the films grown in the 2D-like growth mode are superior to those grown in the island growth mode. It might seem surprising that the films grown in the 2D-like growth mode have inferior physical properties to the films grown in the 3D-to-2D mode since 2D films are usually the highest quality, but the difference in crystallinity between these two films is at the heart of this difference. Both the RHEED patterns and the reciprocal space maps demonstrate that the films grown in the 3D-to-2D growth mode are more crystalline (*i.e.*, possess fewer defects) than the films grown in the 2D-like mode, and this manifests itself in the superior physical properties for these films.

It is important to note that the magnetic moment of the film grown in the 3D-to-2D growth mode compares favorably with Fe_3_O_4_ thin films from previous studies, with it exceeding the magnetic moment of previous Fe_3_O_4_ films less than 100 nm thick grown on SrTiO_3_ by any growth techniques^36^,^37^,^42^,^43^,^44^. The other films in our study (*i*.*e.*, the films grown in the 2D-like and island growth modes) have magnetic properties similar to these previous studies, signifying that these previous studies were likely growing their Fe_3_O_4_ films in these other growth mode regimes. Additionally, the film grown in the 3D-to-2D growth mode has a magnetic moment nearly equal to the magnetic moment of the best Fe_3_O_4_ films less than 100 nm thick grown on MgO with pulsed-laser deposition^28^,^42^,^45^,^46^. The fact that its magnetic moment nearly equals that of films grown on MgO, which has only a 0.3% lattice mismatch with Fe_3_O_4_, demonstrates the high-quality of the films grown in the 3D-to-2D growth mode and the potential for inclusion in perovskite-based devices.

To further investigate how films within a growth mode regime differ from one another, we can look at the *M*-*T* curves [Fig. 5(c)] and *M*-*H* loops [Fig. 6(d)] for three films grown within the 3D-to-2D growth regime. As the laser fluence is increased, systematic changes occur to the magnetic properties of these films. The Verwey transition begins to broaden, with the highest fluence film having a Verwey transition that resembles the best island growth mode film. Additionally the magnetic moment and the coercive field both decrease, along with the hysteresis loops becoming slightly less square. These changes in magnetic properties all point towards an increase in crystalline disorder as the fluence is increased due to increased knock-on damage during growth.

By mapping out the evolution of the structure, morphology, transport properties, and magnetic properties of Fe_3_O_4_ grown on SrTiO_3_ as a function of the energetics of the growth process, we have found a regime where high-quality, thin films of the spinel ferrites can be grown epitaxially and atomically flat on perovskite substrates. These films furthermore have arguably the best magnetic properties observed to date for Fe_3_O_4_ films on SrTiO_3_. The general trend among all three growth modes is that the films with the best physical properties are grown at the lowest fluence possible as long as the films are not too close to the amorphous growth regime boundary. This makes the ideal conditions to grow a sample those which are as close to the 3D-to-2D/2D-like growth regime boundary as possible at the lowest fluence possible since the 3D-to-2D films possess the best physical properties and the boundary between these two growth modes is the lowest fluence at which you can grow a film in the 3D-to-2D growth mode.

### Fe_3_O_4_/La_0.7_Sr_0.3_MnO_3_ spin valve device

**Figure 7:**
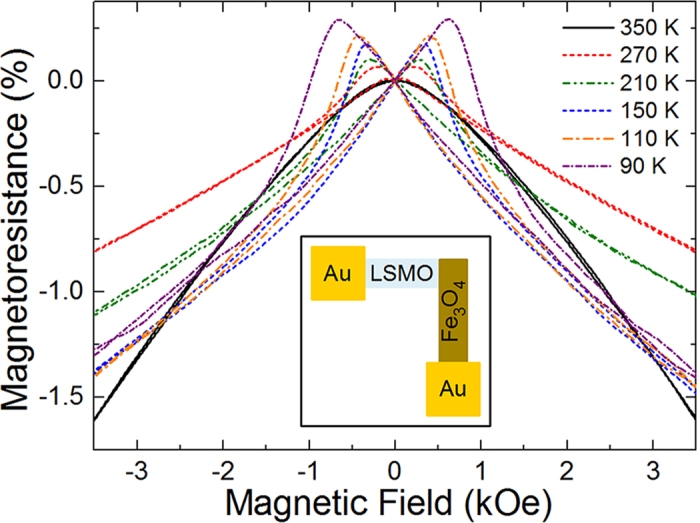
Magnetoresistance for a Fe_3_O_4_/La_0.7_Sr_0.3_MnO_3_/SrTiO_3_ (001) spin valve device with the Fe_3_O_4_ grown at 2.5 J/cm^2^ and 400 °C on the 2D/3D-to-2D phase boundary. Inset shows schematic for La_0.7_Sr_0.3_MnO_3_ (LSMO)/Fe_3_O_4_ spin valve device.

To demonstrate that Fe_3_O_4_ films grown at the 3D-to-2D/2D-like growth mode regime boundary can be incorporated into a real heteroepitaxial device with a functional perovskite material, we grew a 50 nm Fe_3_O_4_/50 nm La_0.7_Sr_0.3_MnO_3_ spin valve device on SrTiO_3_ (001). A La_0.7_Sr_0.3_MnO_3_ wire with dimensions of 1 mm x 200 μm was grown on SrTiO_3_ by using a shadow mask at 2.0 J/cm^2^ at 725 °C in an oxygen pressure of 2×10^−1^ Torr, and following growth the film was cooled in 760 Torr of oxygen. A similar Fe_3_O_4_ wire with the same dimensions was grown at 2.5 J/cm^2^ and 400 °C so that the ends of the two wires overlapped [Inset, Fig. 7]; the low substrate temperature for the growth of Fe_3_O_4_ is necessitated by the fact that Fe_3_O_4_ needs to be grown in vacuum and the La_0.7_Sr_0.3_MnO_3_ quickly reduces when heated at high temperatures in vacuum. We then deposited 50 nm Au films on top of the ends of the Fe_3_O_4_ and La_0.7_Sr_0.3_MnO_3_ wires in order to contact the two leads of the spin valve. The magnetoresistance, defined as 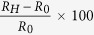, for the spin valve device was measured up to 3.5 kOe at temperatures ranging from 90 K to 350 K [Fig. 7]. At high fields, the magnetoresistance is negative for all temperatures, which is due to the anti-phase boundaries in the Fe_3_O_4_ thin film^47^. At lower fields, however, the magnetoresistance becomes more complicated. At 350 K (near the *T*_C_ of La_0.7_Sr_0.3_MnO_3_), the magnetoresistance is negative for all magnetic field values since we are just measuring the magnetoresistance of the Fe_3_O_4_ film. Below 350 K, where La_0.7_Sr_0.3_MnO_3_ begins to develop a magnetization, as observed by a positive magnetoresistance, the magnetoresistance first increases from a negative value below 0 Oe and becomes positive as the field passes through 0 Oe. For 270 K, the magnetoresistance peaks at ~200 Oe before decreasing and ultimately becoming negative at ~450 Oe. As the temperature is decreased to 90 K, these fields increase to ~600 and ~900 Oe, respectively. The fields where the magnetoresistance changes sign correspond roughly to the coercive fields of La_0.7_Sr_0.3_MnO_3_ and Fe_3_O_4_, leading to a clear explanation for the data. When the field is increased past 0 Oe, the magnetic moment of the La_0.7_Sr_0.3_MnO_3_ layer switches, resulting in the La_0.7_Sr_0.3_MnO_3_ and Fe_3_O_4_ layers having anti-parallel magnetic moments and a larger resistance. Between 450 and 900 Oe (depending on the temperature), the Fe_3_O_4_ layer switches; this is associated with a decrease in resistance due to the magnetic moments in both layers now being parallel. Additionally, the magnetoresistance increases as the temperature decreases due to the enhanced magnetization of the La_0.7_Sr_0.3_MnO_3_ layer. The changes in the magnetoresistance between positive and negative values near the coercive fields of the La_0.7_Sr_0.3_MnO_3_ and Fe_3_O_4_ layers indicate that the Fe_3_O_4_ film is high quality with a sharp interface with La_0.7_Sr_0.3_MnO_3_.

## Conclusions

In summary, we have demonstrated that Fe_3_O_4_ can be grown epitaxially on SrTiO_3_ in three different growth modes, with two of these growth modes, 2D-like and 3D-to-2D, producing films that are atomically flat. Films grown in a 3D-to-2D growth mode have smaller resistivities and larger magnetic moments than films grown in the other two growth mode regimes and are comparable to the best Fe_3_O_4_ films grown on SrTiO_3_ substrates by any method. We have found that the ideal growth conditions to produce the highest quality Fe_3_O_4_ thin film possible on SrTiO_3_ is to grow films at low fluences on the 3D-to-2D/2D-like growth mode regime boundary. By growing a film at these growth conditions epitaxially on a La_0.7_Sr_0.3_MnO_3_ thin film, we created a spin valve device in which the sign of the magnetoresistance displayed distinct features near the coercive fields of La_0.7_Sr_0.3_MnO_3_ and Fe_3_O_4_. This demonstrated that films grown with these growth conditions can be easily incorporated into perovskite based all-oxide devices and should pave the way for more spinel ferrite thin-films to be included into room-temperature, heteroepitaxial, all-oxide devices based on the perovskite crystal system.

## Methods

We grew ~25 nm Fe_3_O_4_ thin films on SrTiO_3_ (001) substrates via pulsed-laser deposition in a background vacuum pressure of 1×10^−6^ Torr. The energy during growth was controlled by adjusting the laser fluence and temperature between 1.2–3.1 J/cm^2^ and 400–600 °C, respectively [Table 1]. Before growth, the substrates were ultrasonically cleaned in acetone and isopropanol and annealed *in situ* at 1×10^−7^ Torr and subsequently at 100 mTorr of O_2_ at the growth temperature for 20 minutes each. The structure and growth mode were monitored during growth with RHEED. Further *ex situ* structural characterization was carried out with high-resolution X-ray diffraction. X-ray reflectivity was used to measure the thickness of each sample, and the surface morphology was measured with atomic force microscopy (AFM) measurements. Transport measurements were performed in a van der Pauw geometry and magnetic measurements were performed with a superconducting quantum interference device (SQUID) magnetometer.

## Additional Information

**How to cite this article**: Moyer, J. A. *et al.* Epitaxial growth of highly-crystalline spinel ferrite thin films on perovskite substrates for all-oxide devices. *Sci. Rep.* **5**, 10363; doi: 10.1038/srep10363 (2015).

## Figures and Tables

**Table 1:** Summary of growth conditions for each sample and the corresponding growth regime, epitaxial strain, and surface roughness.

**Laser fluence (J/cm**^**2**^)	**Substrate temperature (°C)**	**Growth regime**	**Film Thickness (nm)**	**In-plane strain (%)**	**RMS surface roughness (nm)**
1.2	500	Amorphous	25.0	—	0.34 nm
1.5	400	2D-like	29.5	−0.42	0.37 nm
2.3	400	2D-like	29.3	−0.52	0.45 nm
1.5	500	3D-to-2D	24.9	−0.58	0.25 nm
2.3	500	3D-to-2D	26.6	−0.22	0.68 nm
3.1	400	3D-to-2D	23.2	−0.014	1.4 nm
1.2	600	Island	33.2	−0.072	1.2 nm
1.5	600	Island	24.6	−0.018	1.7 nm
3.1	500	Island	17.8	−0.028	2.2 nm
2.3	600	Mixed orientation	30.4	0.0059	3.6 nm
